# Improving the diagnostic efficiency of H1N1 2009 pandemic flu: analysis of predictive clinical signs through a prospective cohort.

**DOI:** 10.1371/currents.RRN1120

**Published:** 2009-10-21

**Authors:** Philippe Brouqui, Vinh Vu Hai, Antoine Nougairede, Jean-Christophe Lagier, Elisabeth Botelho, Laetitia Ninove, Christine Zandotti, Remi N. Charrel, Xavier De Lamballerie, Didier Raoult

**Affiliations:** ^*^Service des Maladies Infectieuses et Tropicales, CHU Nord AP-HM , URMITE CNRS/IRD 6236, faculté de médecine, Université de la méditérranée, Marseille; ^†^Department of Infectious and Tropical Diseases, Viet Tiep hospital, Haiphong, Vietnam; ^‡^Fédération de microbiologie, Assistance publique-hôpitaux de Marseille, Marseille, France ; Unité des virus Emergents, UMR 190 "Emergence des pathologies virales", Université de la Méditerranée & Institut de Recherche pour le Développement, Marseille, France.; ^§^Service des Maladies Infectieuses et Tropicales, Assistance Publique Hopitaux de Marseille, Hôpital Nord, Marseille, France, URMITE, CNRS 6236; ^**^Fédération de microbiologie, Assistance publique-hôpitaux de Marseille, Marseille, France. and ^‡‡^Fédération de microbiologie, Assistance publique-hôpitaux de Marseille, Marseille, France ; Unité de Recherche sur les Maladies Infectieuses et Tropicales Emergentes UMR CNRS 6236 IRD 3R198, IFR 48, Faculté de Médecine, Université de la Méditerranée, Marseille, France.

## Abstract

In late June 2009, we set up a dedicated flu-like illness outpatient consultation in the Infectious Diseases and Tropical Medicine department of Marseille university hospital to detect the new A/H1N1 pandemic influenza and to contain efficiently the A/H1N1 infected patients. For 3 months, we compiled data corresponding to a total of 307 patients who presented with a flu-like syndrome. 31 of them were positive for H1N1 pandemic flu through real-time RT-PCR (rRT-PCR); among them, 19 were positive for a rapid influenza detection test (RIDT). We report here the significant clinical characteristics of A/H1N1 pandemic flu patients compared with other flu-like illnesses, which were used to improve the predictive value of the diagnosis in the current epidemiological situation. We found that regardless of the prevalence of A/H1N1 positive cases in the suspected patients, the absence of cough rejects the diagnosis of A/H1N1 infection in 100% of cases. Among patients referred for flu-like illness, those with cough should be tested for A/H1N1 by RIDT. In the current situation, the PPV and NPV of RIDT for H1N1 reached 90.5% and 95.8 %, respectively. It is important to notice that the 2 RIDT-positive that were negative for H1N1 were seasonal H3N2 influenza indicating that specificity and PPV of RIDT for all influenza was 100%. Therefore, positive RIDT does not require rRT-PCR confirmatory test. Only negative RIDT should be tested with rRT-PCR assay. Respecting this algorithm would have saved up to 70,000 Euros ( 100.000 USD) for the 307 patients and would have resulted in a significant gain of time to transmit the laboratory results to the clinical ward.



**Introduction**

On April 15 and 17, 2009 the US CDC identified two cases of a new A/H1N1 influenza virus characterized by a unique combination of gene segment  from avian, human and swine origin . On May 7 the clinical presentation of the first 642 cases was reported pointing out the prevalence of fever and cough (94 and 92% respectively) and the unexpected presence of diarrhoea and vomiting in 25% of cases each [1] . Since then the WHO declared that a pandemic with the new H1N1 2009 virus was underway. Clinical diagnostic of A/H1N1 pandemic is difficult and two laboratory tests have been released to help clinician in the etiologic diagnostic of this disease. The rapid influenza diagnostic test (RIDT) have been evaluated with a sensitivity of 40-69% depending upon the trade mark and the viral load of the collected sample [2] . The specificity, the PPV and the NPV were evaluated to be 93%, 77%, 94% respectively when compared to real-time Reverse Transcriptase PCR (rRT-PCR) [3] .  The lack of sensitivity of RIDT led to the recommendation to use rRT-PCR test to confirm A/H1N1 pandemic flu diagnosis.

         In France the initial strategy relied on the early identification of suspected cases and containment strategy for the A/H1N1 positive patients in Infectious Disease ward in university referral hospital. This strategy was stopped in July 2009. The estimation of the prevalence of the disease in the population now relies only on the French influenza surveillance system (http://www.grog.org/ ). 

         As reported by the French authorities, during July to September 2009 the prevalence of A/H1N1 pandemic flu among flu-like illness varied from 4% to 10% in August. These data correlates well that obtained in Marseille in our cohort with the point of care strategy [4] . In parallel, the number of influenza like illnesses reported by the French influenza surveillance system was increasing over the outbreak baseline in most of the French region whereas the proportion of microbiologically documented case of A/H1N1 pandemic flu remained stable around a 4% value suggesting that the number of Influenza like illnesses declared was likely due to a raised in practitioner consultation and that the A/H1N1 2009 flu epidemic was not yet declared in our country.

         In September 2009 French news papers reported a patient’s claim that the A/H1N1 rRT-PCR test was charged to the patient himself and not reimburse by the French Social security insurance. The cost of the test being evaluated at 280 Euros (**418 USD**) , it is likely that patients will not accept anymore to be tested for A/H1N1 virus when presenting with flu-like symptoms. This may jeopardize the surveillance of the A/H1N1 based on laboratory diagnosis.

         In this situation we wanted to evaluate through out our cohort of patient seen at our flu-like outpatients clinic, the PPV and NPV of signs and symptoms of the new pandemic flu compared to other flu-like illnesses in order to improve our diagnostic probability. 

**Patients and methods**

Patients who presented a flu-like illness were referred either by their medical practitioner or attended directly the dedicated Flu consultation in our hospital. They were sampled and tested at the point of care (POC) laboratory for RIDT and real-time reverse transcriptase PCR (rRT-PCR) [4]. An epidemiological questionnaire was filled during the time RIDT was performed and the patient returned home if RIDT was negative waiting for the result of the rRT-PCR assay. If a patient presenting with co-morbidity factors [5] was RITD-positive, he was hospitalised and treated with oseltamivir (75mg twice a day for 5 days). If no co-morbidity factors were identified, the patient returned home where confinement was recommended for 7 days with symptomatic treatment. When, 12 hours later, the rRT-PCR returned positive, patients were informed by telephone and those with co-morbidity factors were hospitalised and treated with oseltamivir. The RIDT used in our study was the Directigen™ EZ Flu A+B (Becton-Dickinson, Franklin Lakes, NJ). The rRT-PCR assay was previously described [4] [6]

    The questionnaire was captured with Epidata 3.1, and analysed with Stata 9.2. (StataCorp LP; http://www.stata.com). To establish NPV and PPV we use the Baye’s theorem PPV= Se.p/[Se.p+(1-Sp)(1-p)] and NPV=Sp(1-p)/[Sp(1-p)+(1-Se)p] were Se, Sp, are sensitivity and specificity of the test  and p the prevalence of the disease [7] . We drew the diagrams with Excel® (2007 Microsoft). For the multivariate analysis all variable with a p< 0.25 were included.   

**Results**

From July to September, 307 patients were seen. The weekly prevalence of H1N1 2009 pandemic flu positive patients among all other ranged from 0 to 30% (figure 1). 

**Figure:**
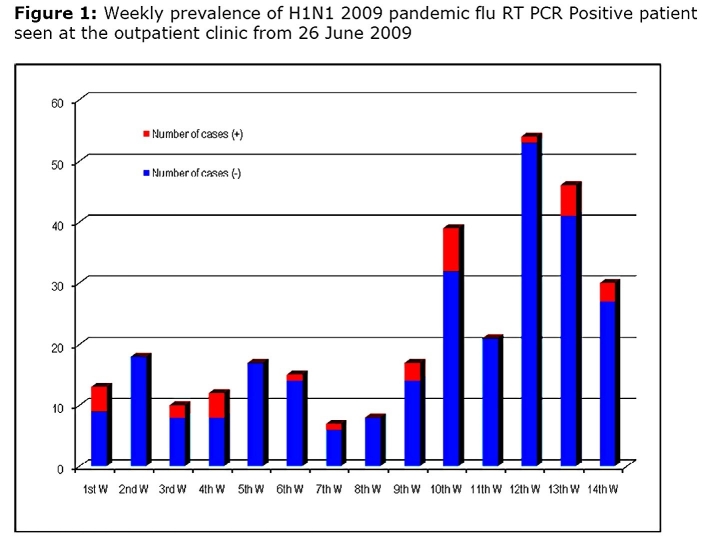

When analysed by univariate analysis, clinical signs and symptoms significantly associated with H1N1 positive group of patient “Flu patient” were cough (31/100%), and diarrhoea (18/48%). Interestingly, myalgia was significantly associated with “non-Flu” patients (20/46%) with a Relative Risk of 0.42. All other clinical signs including fever, asthenia, headaches, and vomiting were not significantly associated with “Flu patient” (Table 1).

**Figure:**
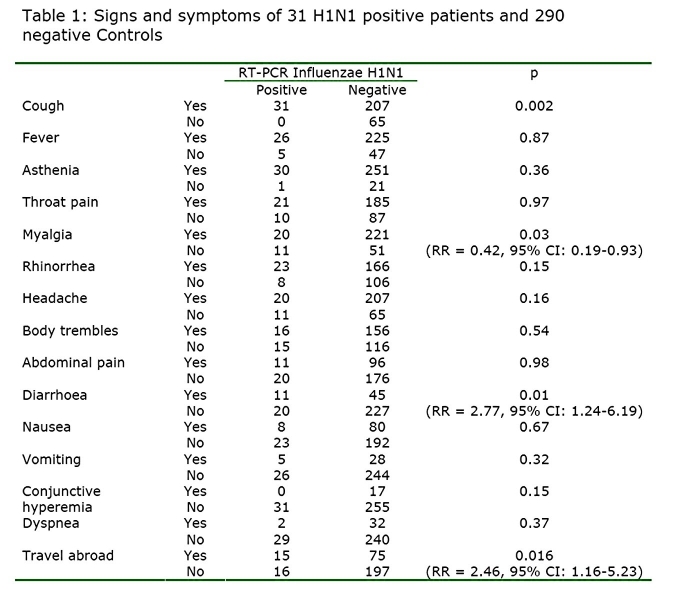

Travel abroad was more frequently associated to “Flu patient” (RR=2.85, 95% CI:1.32-6.17, p=0.006) .  When the variables were included in a multivariate analysis only the presence of diarrhoea was independently associated with “Flu patient” (data not shown).  “Flu patients” (25 ±14 year-old; 95% CI 20.31-30.59) were significantly younger than “non-Flu” patient (31 ±14 year old; 95% CI 29.63-33.17), p=0.035. 

    In our cohort the calculated sensitivity of the RDIT in detecting H1N1 pandemic virus was 61.29%, the specificity 99.28%, the PPV 90.5% and the NPV 95.80 % compared to rRT PCR. In children less than 15 year-old the specificity of RIDT for H1N1 pandemic flu reach 100%. Calculated NPV and PPV related to the prevalence of H1N1 2009 pandemic flu is presented in Figure 2A and 2B.

**Figure:**
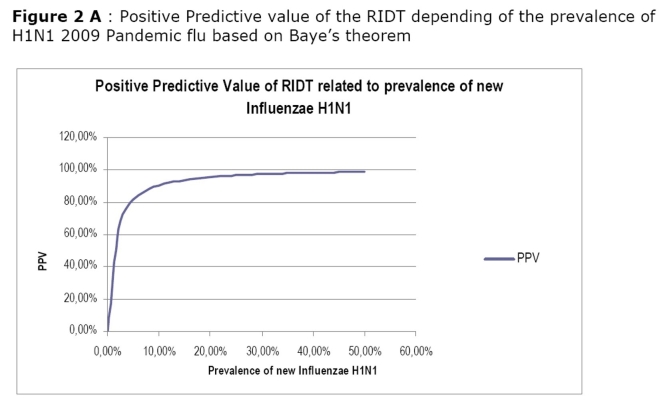

**Figure:**
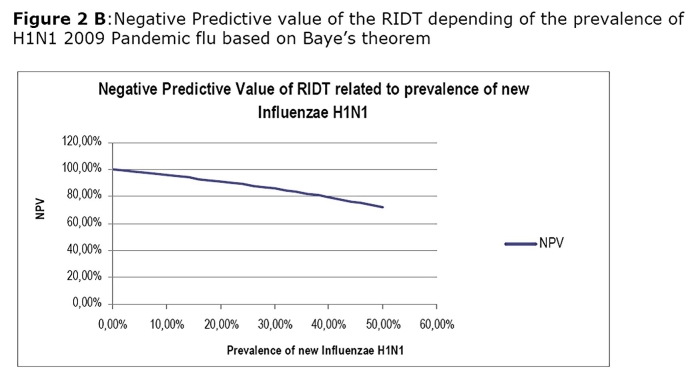

 Two RIDT were positive, the H1N1 specific rRT PCR being negative but these were positive H3N2 seasonal influenza. In these conditions the specificity and the PPV of RIDT for all influenza reach 100%. The calculated sensitivity, specificity, PPV and NPV of cough, absence of myalgia and diarrhoea was 100%, 23.90%, 13.03%, 100%; 35.48%, 81.25%, 17.74%, 91.70% and 35.48%, 83.46%, 19.64%, 91.90% respectively compared to rRT PCR (Table 2). 

**Figure:**
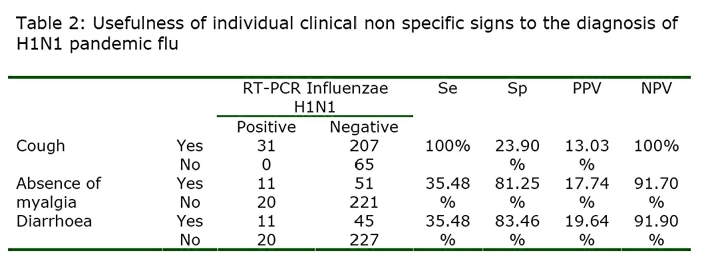

 Calculated NPV and PPV showed that the NPV of cough will stay a 100% whatever the prevalence of the disease will be suggesting that absence of cough will reject the diagnosis (Figure 3 A and B). 

**Figure:**
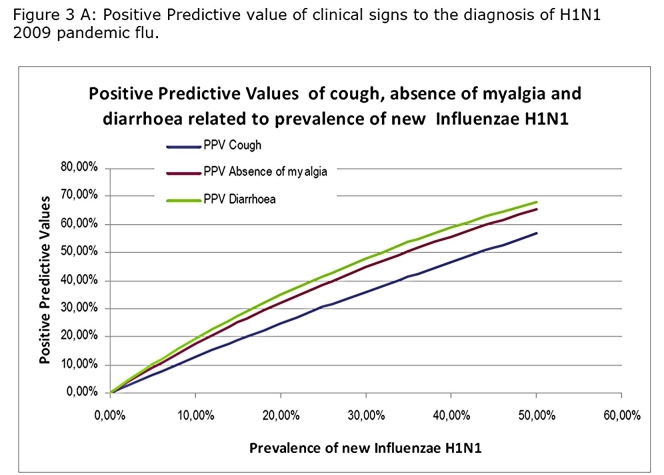

**Figure:**
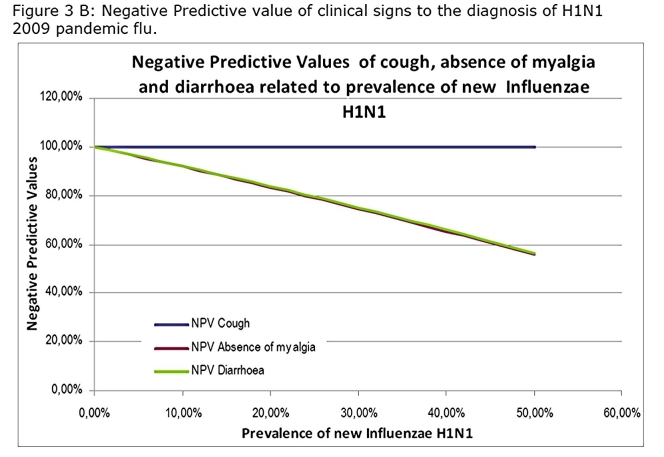

In combining signs, we found that cough and diarrhoea; cough and absence of myalgia; cough, diarrhoea and absence of myalgia were significantly associated with H1N1 positive group of patient (p=0.001, p<0.001 and p=0.020 respectively). The combination between diarrhoea and absence of myalgia was not significantly associated with H1N1 positive group of patient (p=0.057). The calculated sensitivity, specificity, PPV and NPV of these combine signs are reported in the table 3. 

**Figure:**
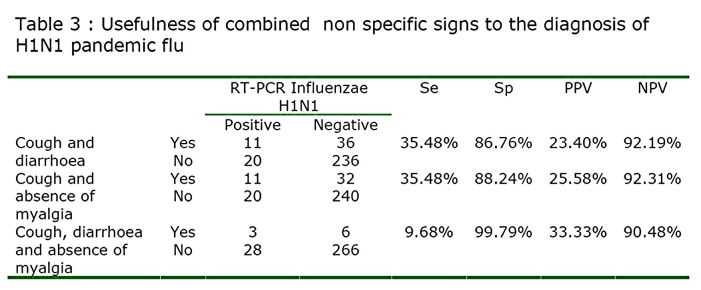

 While association of cough diarrhoea and absence of myalgia rose considerably the PPV especially at high prevalence rate that one does not reach a sufficient level to be clinically useful (Figure 4A ,4B)

**Figure:**
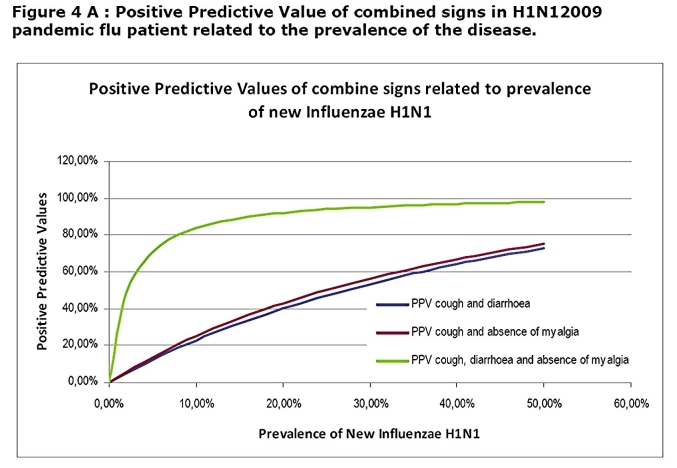

**Figure:**
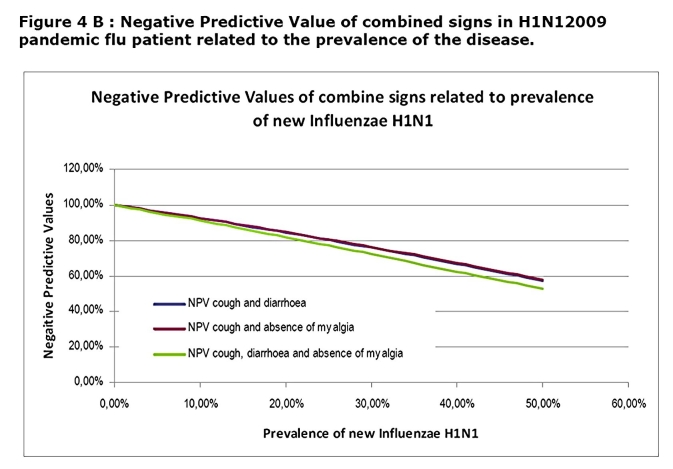

Discussion

A/H1N1 2009 pandemic flu is still ongoing despite the fact that the first wave seems to decrease in some countries such northern Americas and countries of the south hemisphere. Although announced by WHO the second wave is not yet declared and the time remaining to immunize exposed population is not known. In France, as in other European countries, the prevalence of the disease is still low below 10% of flu-like illness. It is still time to try to slow down the progression of the pandemic by reducing human-to-human transmission. In order to do so, the identification of laboratory-confirmed A/H1N1 2009 cases is still needed to better evaluate the need of social distancing measures, to improve the care of exposed persons and those who are at risk such as pregnant women [8] . Diagnostic test are costly and cannot be ordered for all patients presenting with a flu-like illness; this cannot be sustained logistically with the progression of the pandemic and is economically non senses. At the present time (October 20, 2009), in France, a very small proportion of flu-like illnesses are being tested for A/H1N1 virus (i) because of the official guidelines that recommend rRT-PCR testing only for severe cases requiring hospitalization, (ii) and because the rRT PCR test have not been yet disseminated to laboratories other than reference laboratories. As a consequence, the current 8% prevalence of A/H1N1 in suspected patients means that A/H1N1 recommended social distancing measures are prescribed erroneously in 92% of cases, leading to an important economic cost for the society.

         Our study indicates that, in the present situation, a patient who consults for a flu-like illness without cough should be rejected for the diagnosis of A/H1N1 pandemic flu because the negative predictive value is of 100% (Panel 1). 

**Figure:**
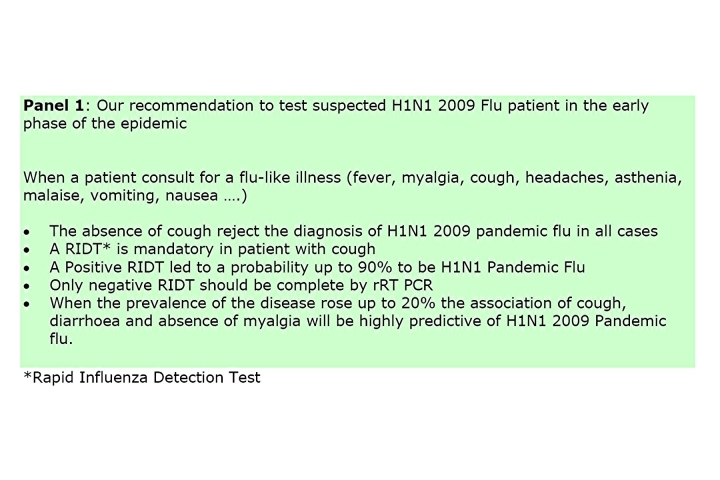

 This will remain true during all the epidemic phase as the NPV will not vary with the prevalence (Figure 3B). In contrast, a patient who present with a flu-like illness and cough should systematically be tested by RIDT because the presence of cough is associated with a sensitivity at 100%. This clinical sign alone will not be predictive of the disease as the PPV is low and will stay low even during the epidemic (Figure 3A). However the association of cough, diarrhoea and the lack of myalgia, although not useful in the early phase, may be useful in the acme of the epidemic as the PPV reaches up to 90% when the prevalence exceeds 20%. 

It is important to notice that RIDT must not be considered as a screening test but as a confirmatory test with a low sensitivity and a high specificity. In patients with flu-like symptoms that were referred to us positive RIDT test provided a PPV at 100% for all influenza and could therefore be sufficient for laboratory confirmation, in contrast with the French recommendations. Finally the rRT-PCR test should be restricted to RIDT negative persons who present with cough. Such recommendation if followed for these 307 patients would have saved 76,440 Euros (**114,307.93 USD**) (31 RIDT and 13 rRT PCR tests). It is important to underline that these results were obtained with a specific RIDT (Directigen, Beckton-Dickinson), and that cannot be transposable to every RIDT manufacturer.

In summary in this early phase of the epidemic, testing for A/H1N1 2009 pandemic virus is necessary to make the more efficient recommendations in term of social distancing measures to slow down as much as possible the epidemic and to optimise the care of patient at risk. Based upon our data RIDT (Directigen, Beckton-Dickinson) which is widely available (and reimbursed by the social security in France) should be prescribed only if cough is present. Negative RIDT samples only should have rRT-PCR tested. Later on, physician should keep in mind that the combination of cough, diarrhoea and the absence of myalgia will be highly predictive of A/H1N1 2009 pandemic flu. 

## Acknowledgments

The authors want to acknowledge Pr H Richet , URMITE CNRS IRD 6236 for statistical help and judicious comments. 

## Funding information

No specific funding have been obtained for this work   

## Competing interests

 The authors have declared that no competing interests exist ****

## References

[ref-3881716193] Novel Swine-origin Influenza A (H1N1) Virus Investigation Team. Emergence of a novel swine-origin Influenza A (H1N1) virus in humans. N.Engl.J.Med. 361, 1-10. 5-7-2009. 10.1056/NEJMoa0903810 19423869

[ref-2853693811] Evaluation of rapid influenza diagnostic tests for detection of novel influenza A (H1N1) Virus - United States, 2009. MMWR Morb Mortal Wkly Rep 2009 August 7;58(30):826-9. 19661856

[ref-3942536288] Ginocchio CC, Zhang F, Manji R, Arora S, Bornfreund M, Falk L et al. Evaluation of multiple test methods for the detection of the novel 2009 influenza A (H1N1) during the New York City outbreak. J Clin Virol 2009 July;45(3):191-5. 10.1016/j.jcv.2009.06.005 PMC7172175 19540158

[ref-1321246912] Antoine Nougairede, Laetitia Ninove, Christine Zandotti, Xavier De Lamballerie, Celine Gazin, Michel Drancourt et al. Point of Care strategy for rapid diagnosis of novel A/H1N1 influenza virus. PLoS Currents Influenza . 9. 10.1371/currents.RRN1039 PMC2762773 20025202

[ref-1866316587] Vaillant L, La RG, Tarantola A, Barboza P. Epidemiology of fatal cases associated with pandemic H1N1 influenza 2009. Euro Surveill 2009;14(33). 10.2807/ese.14.33.19309-en 19712643

[ref-646057932] Ninove L, Gazin C, Gould EA, Nougairede A, Flahault A, Charrel RN et al. A Simple Method for Molecular Detection of Swine-Origin and Human-Origin Influenza A Virus. Vector Borne Zoonotic Dis 2009 June 8. 10.1089/vbz.2009.0110 19505254

[ref-3135195773] Bayes E.T. An assay toward solving a problem in the doctrin of chance. Philos Trans R Soc Lond (Biol) 53, 370-418. 1763.

[ref-821742634] Jamieson DJ, Honein MA, Rasmussen SA, Williams JL, Swerdlow DL, Biggerstaff MS et al. H1N1 2009 influenza virus infection during pregnancy in the USA. Lancet 2009 August 8;374(9688):451-8. 10.1016/S0140-6736(09)61304-0 19643469

